# Supplementary material for: Anti-SARS-CoV-2 Antibody Levels Associated with COVID-19 Protection in Outpatients Tested for SARS-CoV-2, US Flu VE Network, October 2021–June 2022
Source: medRxiv. 2023 Sep 23:2023.09.21.23295919. Preprint. [Version 1] doi: 10.1101/2023.09.21.23295919 (PMC10543239; doi:10.1101/2023.09.21.23295919)
Supplement: Supplement 1 [file NIHPP2023.09.21.23295919v1-supplement-1.pdf]

SUPPLEMENTAL MATERIALS

Supplemental Figure 1. Distribution of anti-RBD and anti-N binding antibody levels across time between symptom onset and dried blood spot collection. Anti-RBD (**S1A**) and anti-N (**S1B**) antibody levels (BAU/mL) by days after reported symptom onset among SARS-CoV-2 rRT-PCR positive patients with and without evidence of prior SARS-CoV-2 infection. Binding antibody levels are presented on the $\log_{10}$ scale. The dotted line represents the manufacturer's cutoff for positivity (≥15.9 BAU/mL for anti-RBD and ≥6.9 BAU/mL for anti-N antibody levels).

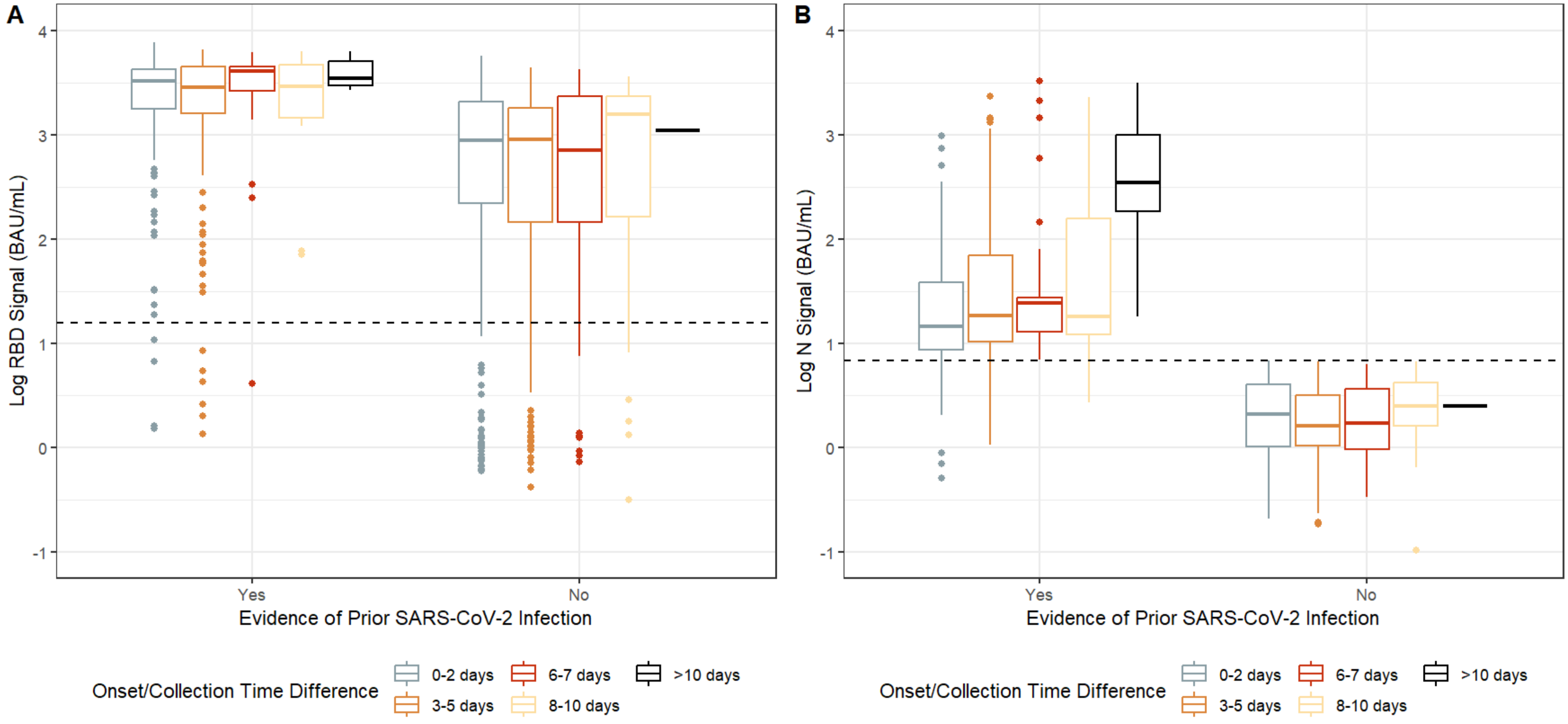

medRxiv preprint doi: https://doi.org/10.1101/2023.09.21.23295919; this version posted September 23, 2023. The copyright holder for this preprint (which was not certified by peer review) is the author/funder, who has granted medRxiv a license to display the preprint in perpetuity. This article is a US Government work. It is not subject to copyright under 17 USC 105 and is also made available for use under a CC0 license.

medRxiv preprint doi: https://doi.org/10.1101/2023.09.21.23295919; this version posted September 23, 2023. The copyright holder for this preprint (which was not certified by peer review) is the author/funder, who has granted medRxiv a license to display the preprint in perpetuity. This article is a US Government work. It is not subject to copyright under 17 USC 105 and is also made available for use under a CC0 license.

Supplemental Figure 2. Distribution of anti-RBD and anti-N binding IgG antibody levels across COVID-19 case and vaccination status. Anti-RBD (**S2A**) and anti-N (**S2B**) antibody levels (BAU/mL) COVID-19 case and test-negative control status and number of COVID-19 vaccine doses received. Binding antibody levels are presented on the $\log_{10}$ scale. The dotted line represents the manufacturer's cutoff for positivity (≥15.9 for anti-RBD and ≥6.9 for anti-N antibody levels).

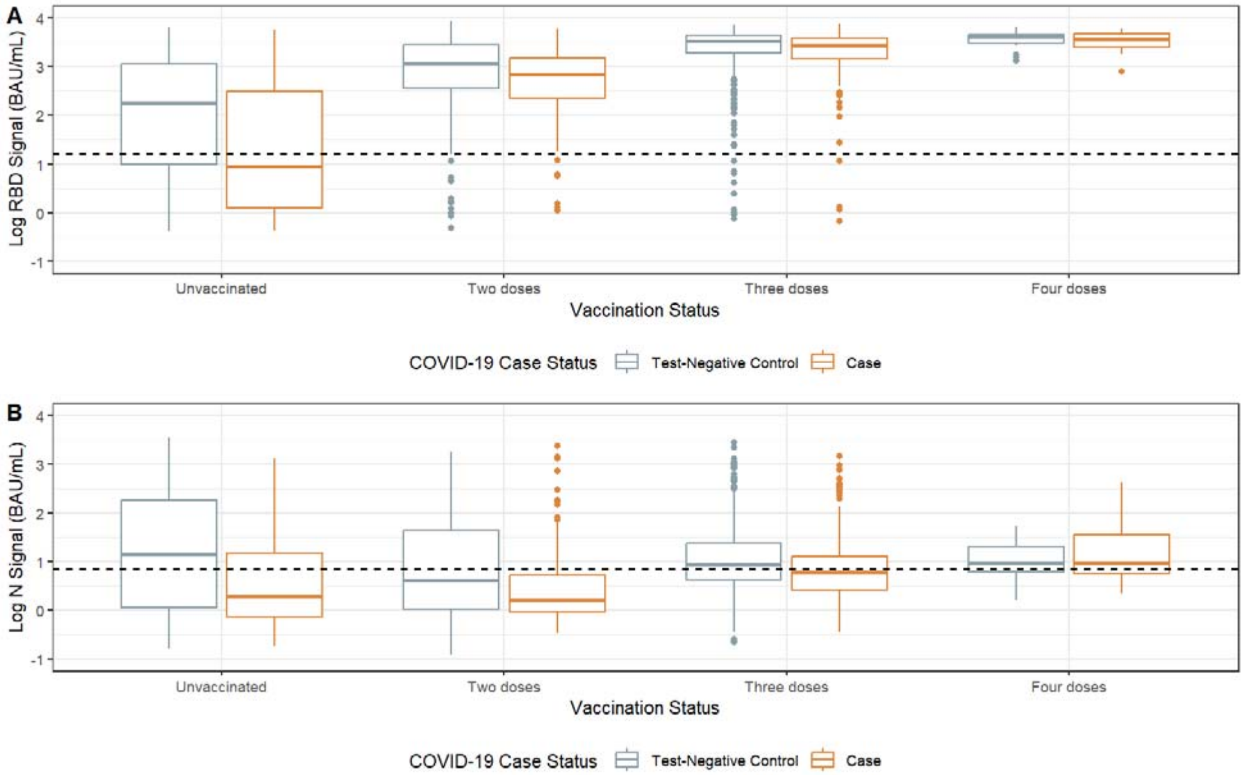

Supplemental Table 1. Assessment of potential confounding covariates for association between anti-RBD binding antibody levels and symptomatic COVID-19 during the Omicron variant period

**Covariate assessed**	**Total number of observations**	**β[1]**	**Percent change in estimate from original adjustment set[2]**	**AIC**	**Log-likelihood test *p*-value[2]**
Original adjustment set[3]	1448	-2.1×10^{-4}	—	1826.7	—
Variables removed from original adjustment set:					
**- COVID-19 vaccination status**	1448	-2.3×10^{-4}	**9.0%**	1824.1	0.340
**- Age (cubed)**	1448	-2.2×10^{-4}	4.3%	1845.9	**<0.001**
- Sex	1450	-2.1×10^{-4}	0.1%	1826.7	NA[4]
- Race-ethnicity	1466	-2.1×10^{-4}	0.9%	1851.3	NA[4]
**- Study site**	1448	-2.0×10^{-4}	3.9%	1848.6	**<0.001**
**- Illness onset week**	1448	-2.0×10^{-4}	**5.4%**	1836.5	**<0.001**
- Presence of chronic medical condition	1494	-2.0×10^{-4}	4.5%	1891.5	NA[4]
**- High-risk SARS-CoV-2 exposure**	1448	-2.1×10^{-4}	1.0%	1837.9	**<0.001**

Abbreviations: AIC, Akaike information criterion
[1] The β estimate for the association between anti-RBD binding antibody levels (coded linearly) and odds of COVID-19 illness is shown.
[2] Covariates that when added changed the β estimate by >5% or had a *p*-value <0.05 by the log-likelihood ratio test are bolded.
[3] Original adjustment set included COVID-19 vaccination status, age, sex, race-ethnicity, study site, illness onset week, presence of at least one chronic medical condition, and high-risk SARS-CoV-2 exposure.
[4] Not applicable because different total number of observations than adjusted model.

medRxiv preprint doi: https://doi.org/10.1101/2023.09.21.23295919; this version posted September 23, 2023. The copyright holder for this preprint (which was not certified by peer review) is the author/funder, who has granted medRxiv a license to display the preprint in perpetuity. This article is a US Government work. It is not subject to copyright under 17 USC 105 and is also made available for use under a CC0 license.

Supplemental Table 2. Fifty percent threshold for reduced odds of symptomatic COVID-19 stratified by doses of COVID-19 vaccine received

Variable	Delta period[1]		Omicron period[2]	
	No. cases/Total (%)	50% threshold (BAU/mL)[3]	No. cases/Total (%)	50% threshold (BAU/mL)[3]
**COVID-19 vaccination status**				
Unvaccinated	28/110 (25%)	696	85/245 (35%)	2943
2 doses	45/279 (16%)	3128	150/383 (39%)	1712
3 doses	14/114 (12%)	961	326/847 (38%)	8528
4 doses	—	—	14/40 (35%)	2680

Abbreviations: N, Nucleocapsid; RBD, receptor binding domain
[1] The Delta-predominant period was defined as the period from October 1–December 24, 2021.
[2] The Omicron-predominant period was defined as the period from December 25, 2021–June 29, 2022.
[3] Percent reduction in symptomatic COVID-19 was estimated by (1-adjusted odds ratio) x 100, using the adjusted odds ratio produced by a logistic regression model adjusted for age, study site, illness onset week, and high-risk SARS-CoV-2 exposure. The 50% threshold was where the percent reduction curve crossed 50%.

medRxiv preprint doi: https://doi.org/10.1101/2023.09.21.23295919; this version posted September 23, 2023. The copyright holder for this preprint (which was not certified by peer review) is the author/funder, who has granted medRxiv a license to display the preprint in perpetuity. This article is a US Government work. It is not subject to copyright under 17 USC 105 and is also made available for use under a CC0 license.
